# Ringing the bell for quality P.E.: What are the realities of remote physical education?

**DOI:** 10.1093/eurpub/ckac082

**Published:** 2022-08-26

**Authors:** Viktoria A Kovacs, Tamas Csanyi, Rok Blagus, Mirko Brandes, Gregor Starc, Paulo Rocha, Claude Scheuer, Anthony D Okely

**Affiliations:** Hungarian School Sport Federation, Budapest, Hungary; Hungarian School Sport Federation, Budapest, Hungary; Faculty of Primary and Pre-School Education, ELTE Eotvos Lorand University, Budapest, Hungary; Institute of Teacher Education, Hungarian University of Sports Science, Budapest, Hungary; Institute for Biostatistics and Medical Informatics, Medical Faculty, University of Ljubljana, Ljubljana, Slovenia; Leibniz Institute for Prevention Research and Epidemiology (BIPS), Bremen, Germany; University of Ljubljana, Ljubljana, Slovenia; Portuguese Institute of Sport and Youth, Lisbon, Portugal; University of Luxemburg, Esch-sur-Alzette, Luxembourg; Early Start and Illawarra Health & Medical Research Institute, University of Wollongong, Australia

## Abstract

**Background:**

To date, few data on the quality and quantity of online physical education (P.E.) during the COVID-19 pandemic have been published. We assessed activity in online classes and reported allocated curriculum time for P.E. in a multi-national sample of European children (6–18 years).

**Methods:**

Data from two online surveys were analysed. A total of 8395 children were included in the first round (May–June 2020) and 24 302 in the second round (January–February 2021).

**Results:**

Activity levels during P.E. classes were low in spring 2020, particularly among the youngest children and in certain countries. 27.9% of students did not do any online P.E. and 15.7% were hardly ever very active. Only 18.4% were always very active and 14.9% reported being very active quite often. In winter 2020, we observed a large variability in the allocated curriculum time for P.E. In many countries, this was lower than the compulsory requirements. Only 65.7% of respondents had the same number of P.E. lessons than before pandemic, while 23.8% had less P.E., and 6.8% claimed to have no P.E. lessons. Rates for no P.E. were especially high among secondary school students, and in large cities and megapolises.

**Conclusions:**

During the COVID-19 pandemic, European children were provided much less P.E. in quantity and quality than before the pandemic. Countermeasures are needed to ensure that these changes do not become permanent. Particular attention is needed in large cities and megapolises. The critical role of P.E. for students’ health and development must be strengthened in the school system.

## Introduction

Reaching adequate levels of physical activity throughout childhood provides benefits to physical, physiological and psychological health.^1^ In recognition of these benefits, guidelines recommend that school-aged children should accumulate an average of at least 60 min per day of moderate- to vigorous-intensity physical activity (MVPA) for healthy physical and psychological development.^2^ Physical education (P.E.) classes provide opportunities for all children to engage in regular and developmentally appropriate physical activity and can contribute, at least partly, to meeting the physical activity guidelines. P.E. classes play an important role in developing physical literacy that develop competencies for life-long physical activity and for maintaining appropriate levels of physical fitness.^3^^,^^4^ Moreover, P.E. classes have the potential to improve students’ academic performance and cognition.^5^ Finally, if classes are appropriately facilitated, P.E. can support the development of social skills and social behaviours.^6^

In March 2020, the COVID-19 pandemic started its impact across Europe. Opportunities for physical activity significantly decreased among children in many countries because of the introduced countermeasures.^7^ Social isolation and consequent stress added to functional and cognitive impairments among children.^8^^,^^9^ In the presence of wide scale school closures, online P.E. classes were one of the solutions to provide a meaningful contribution to children’s physical activity, and to possibly mitigate some of the health and mental health consequences such as unhealthy weight gain, and increased rates of depression and anxiety. While research concerning teachers’ perspectives on remote P.E. provision has been reported,^10^ much remains unknown regarding students’ participation in P.E. lessons and allocated curriculum time. Thus, the purpose of this article was to report the level of activity and time spent in online P.E., and to recommend potential avenues for improvement in the post-pandemic period using data from two data collection rounds of a cross-sectional study in 10 European countries.

## Methods

This study was based on secondary analyses of our previously obtained data.^7^^,^^11^ The primary aim of the repeated cross-sectional study was to examine physical activity and screen time in a large multi-national sample of European school children during different time points of the COVID-19 pandemic, to understand which factors may be associated with adherence to physical activity and screen time guidelines, as well as to analyse changes in physical activity and screen time between the different time points. Two online surveys were distributed 8 months apart in spring 2020 and winter 2020. The main characteristics of the two rounds are presented in table 1. Data collection methods were standardized across countries. For older children, the questionnaire was self-administered. Parents of younger children were asked to help their child complete the questionnaire.

**Table 1:** Main characteristics of the two cross-sectional data collection rounds

	Round 1—Spring 2020	Round 2—Winter 2020
Data collection period	15 May and 22 June 2020	26 January and 26 February 2021
Survey open (days)	13 to 23	7 to 18
Participating countries	*France*, Germany, Hungary, Italy, Poland, Portugal, *Romania*, the Russian Federation, Slovenia, Spain (*n* = 10)	*Denmark*, Germany, Hungary, Italy, Poland, Portugal, the Russian Federation, Slovenia, Spain (*n* = 9)
Target recruitment	At least 200 children (6 to 18 years) per country	At least 200 children (6 to 18 years) per country
Sampling	Convenience sampling	Convenience sampling
Survey instrument	20-item online questionnaire assessing total screen time, physical activity (PAQ(C), single item PAQ) and correlates of PA	37-item online questionnaire assessing academic and recreational screen time, physical activity (single item PAQ), P.E. provision, organized sports participation, changes in PA compared to the pre-COVID period and demographic data
Number of all respondents / final analytic sample (*n*)	8997 / 8395	28 273 / 24 302
Median age (IQR) (years)	13 (10–15)	12 (9–14)
Ethical approval	Scientific and Research Ethics Committee of the Medical Research Council in Hungary (Approval Number: IV/5613-3/2020/EKU), and by each centre’s local ethics committee	Scientific and Research Ethics Committee of the Medical Research Council in Hungary (Approval Number: IV/307-1/2021/EKU), and by each centre’s local ethics committee

PA, physical activity; PAQ(C), Physical Activity Questionnaire for Children.

To assess activity level in online P.E. classes, we used one of the PAQ(C) items from round 1.^12^ Specifically, the respondents were asked, ‘In the last 7 days, during your online physical education (P.E.) classes, how often were you very active (playing hard, running, jumping, throwing, strengthening)?’. Response options were ‘I don’t do any online P.E.’, ‘Hardly ever’, ‘Sometimes’, ‘Quite often’ or ‘Always’. This question, together with other PAQ(C) items, was only included in round 1.

To gather quantitative information about P.E. lessons, we analysed two questions from round 2. Respondents reported the number of provided P.E. lessons (both in-school and online) in the last 7 days. Response options ranged from zero to more than 5 lessons (one lesson unit was set in 45 min). Then participants were asked whether the current number of P.E. lessons (both in-school and online) is less or more than they usually have compared to the pre-pandemic period. Response options were ‘It is more’, ‘It is the same’, ‘It is less’ or ‘There is no P.E. lesson at all’. We also collected information from each country coordinator about the number of compulsory P.E. lessons and about the state of school closures in their country. Questions regarding socio-economic and demographic data were taken from the WHO Childhood Obesity Surveillance Initiative.^13^

Survey data were obtained from the online platforms, pooled and cleaned. Data were reported as frequencies and percentages (%) with 95% confidence intervals (CIs), calculated using normal approximation with continuity correction, for categorical variables and quartiles with 95% CIs, calculated using the method proposed by Nyblom,^14^ for numeric variables. The pair-wise association was tested with chi-squared tests with Yates continuity correction. The association was verified also with ordinal logistic regression, considering the frequency of the P.E. (round 1) and the number of P.E. lessons in comparison to before the pandemic (round 2) as dependent variables in two separate models. These results were reported as odds ratios (ORs) with respective 95% CIs. The same model was used in the multivariate analysis, where sex, age, residence and country of residence were included in the model as independent variables for round 1 of the survey. For round 2, sex, age, residence, country of residence and socio-economic status (SES) were included in the model as independent variables. R language for statistical computing was used for the analyses (R version 4.0.5; R Core Team, Vienna, Austria). All reported CIs and *P*-values were two-sided. A *P*-values of <5% was considered statistically significant.

## Results

In round 1, even if P.E. lessons were provided, more than one-fourth of students claimed that they did not do any online P.E. and another 15.7% declared that they were hardly ever very active during the online classes. Slightly less than 20% stated that they were always very active and an additional 14.9% reported being very active quite often (table 2).

**Table 2:** Proportion of children who claimed not doing any online P.E., or being hardly ever or always very active during the online P.E. lessons in May–June 2020 (*n* = 8395 primary and secondary school children)

	‘I did not do any online P.E. in the last 7 days’	‘I was hardly ever very active during online P.E. in the last 7 days’	‘I was always very active during online P.E. in the last 7 days’	Univariate OR^a^ (95% CI)	Multivariate OR^b^ (95% CI)
All, *n* = 8395	27.9	15.7	18.4		
Sex				*P* = 0.038	*P* = 0.602
Boys (46.9)	29.4	15.6	17.8	Ref.	Ref.
Girls (53.1)	26.5	15.8	19.0	1.1 (1.0–1.2)	1.0 (0.9–1.1)
Age				*P* < 0.001	*P* < 0.001
6–10 years (26.9)	38.3	12.6	15.9	Ref.	Ref.
11–14 years (45.1)	20.8	16.8	22.2	1.8 (1.6–2.0)	1.3 (1.1–1.4)
15–18 years (27.9)	29.3	16.9	14.9	1.2 (1.1–1.4)	1.0 (0.8–1.2)
Residence				*P* < 0.001	*P* < 0.001
Capital (1.9)	35.5	17.9	11.5	Ref.	Ref.
City (47.6)	33.1	15.5	12.6	1.2 (1.0–1.4)	1.3 (1.1–1.6)
Rural area or village (42.4)	20.3	15.4	26.6	2.1 (1.8–2.5)	1.6 (1.4–1.9)
Country of residence				*P* < 0.001	*P* < 0.001
France (2.5)	76.1	7.7	1.0	Ref.	Ref.
Germany (2.9)	95.0	2.5	0	(0–0.3)	0.1 (0.0–0.3)
Hungary (31.3)	27.9	11.5	13.0	9.0 (6.5–12.6)	8.5 (6.2–11.9)
Italy (2.9)	50.4	14.6	8.3	3.3 (2.2–5.1)	3.1 (2.0–4.7)
Poland (6.2)	46.9	16.6	8.0	3.5 (2.4–5.1)	3.6 (2.5–5.2)
Portugal (13.8)	14.0	25.8	140.0	10.3 (7.4–14.6)	10.1 (7.2–14.2)
Romania (3.5)	19.4	17.4	13.6	10.6 (7.3–15.5)	9.5 (6.5–14.0)
Russian Federation (3.8)	55.9	21.3	3.2	2.2 (1.5–3.3)	2.2 (1.4–3.3)
Slovenia (22.6)	13.4	17.5	43.7	20.1 (14.5–28.3)	16.2 (11.6–22.9)
Spain (10.7)	23.0	14.2	11.5	9.6 (6.7–13.6)	9.8 (7.0–14.0)

*Note*: Data in the first three columns are reported as relative frequencies.

For question ‘In the last 7 days, during your online physical education (P.E.) classes, how often were you very active (playing hard, running, jumping, throwing, strengthening)?’ we presented relative frequencies only for the three most relevant response options (‘I don’t do any online P.E.’, ‘Hardly ever’, or ‘Always’) and we did not indicate the results for the two other response options (‘Sometimes’ and ‘Quite often’).

a Odds ratio for being more active during the online P.E. lessons as estimated by the univariate ordinal regression. The reported *P*-values are from a Chi-squared test with Yates continuity correction.

b Odds ratio for being more active during the online P.E. lessons as estimated by the multivariate ordinal regression adjusted for all other variables presented in the table. The reported *P*-values are from a likelihood ratio test.

After adjusting for other variables, there were no gender differences, however, children aged 11–14 years reported being more active than other children. Children living in rural area or villages claimed to be more active than their urban peers [multivariate OR 1.6 (95% CI 1.4–1.9)]. Slovenian children reported being more active than children from other countries (table 2).

In the second wave in January–February 2021, we observed that, compared with the obligatory curriculum requirements, the actual number of P.E. lessons was lower in each country, except for the Russian Federation and some parts of Spain and Slovenia (table 3). The actual number of P.E. lessons was low in Germany, Italy and Denmark. This was in line with what was reported by the respondents regarding the change in the number of P.E. lessons (table 4). Around two-thirds of children stated that they had the same number of P.E. lessons than before the pandemic (65.7%), while around one-quarter claimed to have less P.E. lessons (23.8%), and 6.8% said that there were no P.E. lessons at all.

**Table 3:** Comparison of compulsory and actual P.E. curriculum time allocation in nine European countries in January–February 2021 (*n* = 24 302 primary and secondary school children)

Country	Type of education	Number of compulsory P.E. lessons (per week)	Actual number of P.E. lessons (per week)		
			Median (% difference^a^)	Q1 (% difference^a^)	Q3 (% difference^a^)
Denmark (*n* = 1150)	Online	5	1 (−80)	0 (−100)	2 (−60)
Germany (*n* = 1203)	Online	2–3^b^	0 (−100)	0 (−100)	1 (−66.7 to −50)
Hungary (*n* = 857)	Primary: in person Secondary: online	5	4 (−20)	2 (−60)	5 (0)
Italy (*n* = 617)	Varied^b^	2	1 (−50)	1 (−50)	2 (0)
Poland (*n* = 1990)	Primary: in person Secondary: online	5	3 (−40)	2 (−60)	4 (−20)
Portugal (*n* = 458)	Online	3–3.5^b^	2 (−42.8 to −33.3)	1 (−71.4 to −66.7)	3 (−14.3 to 0)
Russian Federation (*n* = 11 686)	In person	2	3 (50)	2 (0)	3 (50)
Slovenia (*n* = 5642)	Primary: in person Secondary: online	2–3^b^	2 (−33.3 to 0)	1 (−66.7 to −50)	3 (0 to 50)
Spain (*n* = 699)	In person	2–3^b^	2 (−33.3 to 0)	2 (−33.3 to 0)	3 (0 to 50)

a Relative difference was calculated as (actual-compulsory)/compulsory*100%. For countries with an interval for compulsory P.E. lessons, we considered both endpoints of the interval.

b Variations exist between regions.

**Table 4:** Proportion of children who reported no P.E. lessons or having fewer, the same or more P.E. lessons than before the COVID-19 pandemic in January–February 2021 (*n* = 24 302 primary and secondary school children)

	No P.E. lessons provided	‘The current number of P.E. lessons is less’	‘The current number of P.E. lessons is the same’	‘The current number of P.E. lessons is more’	Univariate OR^a^ (95% CI)	Multivariate OR^b^ (95% CI)
All, *n* = 24 302	6.8	23.8	65.7	3.7		
Sex					*P* = 0.023	*P* = 0.152
Boys (51.6)	7.2	24.1	65.1	3.6	Ref.	Ref.
Girls (48.3)	6.4	23.5	66.3	3.8	1.1 (1.0–1.2)	1.1 (0.9–1.2)
Age, *n* = 24 297					*P* < 0.001	*P* < 0.001
6–10 years (39.3)	7.2	19.1	69.5	4.2	Ref.	Ref.
11–14 years (36.6)	5.7	24.1	67.3	3.0	0.9 (0.8–0.9)	0.8 (0.7–0.9)
15–18 years (24.1)	7.9	31.0	57.0	4.0	0.6 (0.5–0.7)	0.8 (0.7–0.9)
Residence					*P* < 0.001	*P* < 0.001
Megapolis (8.2)	18.6	19.6	59.5	2.3	Ref.	Ref.
Large city (27.3)	8.3	26.3	62.7	2.3	1.4 (1.2–1.5)	1.1 (0.9–1.2)
Town (20.9)	5.2	22.6	68.3	3.8	1.9 (1.6–2.1)	1.1 (1.0–1.3)
Small town (16.9)	4.5	24.9	66.6	4.0	1.8 (1.6–2.1)	1.1 (0.9–1.3)
Rural area or village (26.7)	4.4	22.8	67.9	4.9	2.0 (1.8–2.3)	1.4 (1.2–1.6)
Socio-economic status, *n* = 23 022					*P* < 0.001	*P* = 0.002
Easily pass the month (34.5)	12.1	28.8	54.9	4.2	Ref.	Ref.
Pass the month w.o. issues (47.4)	3.8	21.1	71.6	3.5	2.0 (1.8–2.1)	1.0 (0.9–1.1)
Trouble making it through (14.5)	4.4	16.6	75.5	3.5	2.4 (2.2–2.6)	0.8 (0.7–0.9)
Barely making it through (3.7)	5.1	22.1	69.2	3.7	1.8 (1.5–2.2)	0.8 (0.7–1.0)
Country of residence					*P* < 0.001	*P* < 0.001
Denmark (4.7)	0	68.2	25.6	6.2	Ref.	Ref.
Germany (5.0)	62.5	29.8	7.1	0.7	0.1 (0.0–0.1)	0.1 (0.0–0.1)
Hungary (3.5)	5.5	33.6	58.5	2.5	2.2 (1.8–2.7)	2.7 (2.2–3.3)
Italy (2.5)	19.1	36.0	42.1	2.8	0.9 (0.7–1.2)	0.9 (0.7–1.2)
Poland (8.2)	9.3	27.0	62.3	1.5	2.3 (2.0–2.7)	2.5 (2.1–2.9)
Portugal (1.9)	6.6	34.3	58.1	1.1	2.0 (1.6–2.5)	2.1 (1.6–2.6)
Russian Federation (48.1)	0.6	7.3	88.9	3.2	11.3 (10.0–12.1)	10.9 (9.5–12.5)
Slovenia (23.2)	7.9	44.0	41.9	6.2	1.4 (1.2–1.6)	1.3 (1.1–1.6)
Spain (2.9)	0.7	13.9	80.6	4.9	8.4 (6.7–10.4)	8.3 (6.5–10.5)

*Note:* Numbers reported in the first five columns are relative frequencies.

a Odds ratio for having more P.E. lessons relative to before the pandemic as estimated by the univariate ordinal regression. The reported *P*-values are from a Chi-squared test with Yates continuity correction.

b Odds ratio for having more P.E. lessons relative to before the pandemic as estimated by the multivariate ordinal regression adjusted for all other variables presented in the table. The reported *P*-values are from a likelihood ratio test.

The proportion of those children who claimed less allocated time for P.E. increased with age (6–10 years: 19.1% vs. 11–14 years: 24.1% vs. 15–18 years: 31%), while the lack of P.E. lessons was more frequent among those living in large cities or megapolises (rural area, village or small town: 4.4% vs. large city: 8.3% vs. megapolis: 18.6%). These differences remained significant after adjusting for all other factors. Also, the proportion of those respondents who claimed that P.E. lessons were not provided was more than two times higher among the highest SES group compared to the lowest SES group (12.1% vs. 5.1%, respectively). However, this difference disappeared after adjusting for the place of residence and the other variables. Almost two-thirds of German children and one-fifth of Italian children reported a lack of P.E. provision (62.5% and 19.1%, respectively), while the proportion of children who reported a lower number of P.E. lessons was the highest in Denmark (68.2%). No differences were observed between boys and girls (table 4).

## Discussion

Our findings reinforce previous observations that P.E. was less optimal during distance learning compared with face-to-face classes. We found low activity levels during the online lessons, particularly among the youngest children and in certain countries. We also observed that the actual time allocated for P.E. was less than that the compulsory requirements, especially in Germany, Italy and Denmark. The low allocated curriculum time was overall more prominent among secondary school students, and in large cities and megapolises. The observed low number of P.E. lessons is particularly worrying, as at the time of our data collection many countries had already eased COVID restrictions and children were allowed to attend school in person. This may indicate a loss of many of the potential benefits of P.E. in the post-pandemic period, especially in assisting students in acquiring the knowledge, neuromotor skills, attitude and confidence to enjoy a lifetime of healthful physical activity. There is a need to ensure that these changes do not become permanent.

In the last 2 years, many students have been affected by the unprecedented closure of school premises, with potential long-lasting impacts which extend beyond the academic effects.^15^ Although abundant teaching resources have been provided, the implementation of distance teaching in P.E. faced many challenges, such as the lack of digital literacy of teachers, the difficulty in implementing conventional teaching plans online, the limited conditions for students to exercise at home, and overall doubts about the importance of online P.E.^16^ Different teachers used different approaches to ensure the continuity of P.E. such as online classes with live streaming, recorded videos, tasks for students, projects or just links to follow.^17^ However, it seems that despite these efforts, in most cases the quantity of P.E. lessons was low.

Among all participating countries, the actual number of online P.E. lessons was the lowest in Germany. On the one hand, it has to be noted that by the time of the data collection, schools were closed and only online education was provided in Germany. However, in other countries with same situation such as Denmark and Portugal, around 32% and 70% of the compulsory P.E. lessons were successfully delivered during distance teaching. Presumably, teachers and/or school authorities in Germany considered P.E. as a less important subject in comparison to others, and weighted the advantages and disadvantages of P.E. provision differently to other countries. Interestingly, renowned German institutions, such as the German Society for Epidemiology, the German Society for Public Health, the German Society of Pediatrics and Adolescent Medicine and the German Society of Pediatric Infectology jointly developed a S3-guideline^18^ and clearly pointed out that the positive effects of P.E. classes in schools predominate. Consequently, the S3-Guideline has to be quickly distributed on the policy level, and school authorities and teachers in Germany have to increase their efforts to maintain P.E. classes, even under pandemic conditions.

The claimed decrease in the allocated curriculum time among secondary school students, as well as in large cities and megapolises during the pandemic is a significant issue as it is known that activity levels are lower among adolescents,^19^ and among those who are living in more densely populated areas.^20^ If delivered, P.E. can be seen as a large-scale high-reach physical activity intervention. Although the number of P.E. classes seems to have fallen the most among those who would need it the most.

The decreased level of physical activity during the pandemic already has measurable consequences on physical fitness^21^^,^^22^ and on the prevalence of obesity^22^ in children. For instance, in Slovenia, the level of physical fitness declined for more than 13% and affected over 70% of the child population of 6–14 years, while the obesity rates increased for more than 20%.^23^^,^^24^ This drastic deterioration occurred despite the very fast and effective response of schools and experts who published special COVID-19 physical activity recommendations only one day after the pandemic was declared,^25^ and despite children’s high engagement in online P.E. classes which was considerably higher than their peers’ from other participating countries. We also have to note that Slovenia was one of the few countries where the allocated curriculum time for P.E. has not decreased during the pandemic compared to the compulsory requirements. Therefore, the losses in physical fitness in the other European countries are probably even more pronounced than in Slovenia.

The low level of physical activity along with the high prevalence obesity among children and adolescents was already concerning before the pandemic.^26^^,^^27^ P.E. provides opportunities to address these public health challenges, particularly as it is an available measure for all children. Accordingly, quality P.E. is a major part of UNESCO’s recommended response to the COVID-19 pandemic.^28^ It has to be noted, however, that due to pedagogical, individual and environmental factors students spend on average less than half of the available time in MVPA during P.E. classes.^29^^,^^30^ Therefore, P.E. classes alone are not the only approach to support children in meeting the physical activity guidelines and to realize health benefits. There is a long-standing debate about how P.E. content influences out-of-school movement behaviours, especially in secondary schools. Studies have highlighted the importance of providing greater choice and a wider range of lifetime physical activities (e.g. health-related exercises and sport activities) that can be easily transferred into the out-of-school settings and into adulthood.^31^^,^^32^ In addition to the content elements of the curriculum, it is necessary to rethink how they will be implemented in the future. P.E. curriculum content that is useful, applicable and relevant to everyday life, and increases physical literacy, should become more dominant in a more meaningful pedagogical environment. In older children and adolescents, individual goal setting and self-monitoring skills should be more emphasized in the future, especially in sport-oriented curriculum models. It is recommended to implement all this through the combined use of easily accessible and widely used digital devices and applications (e.g. mobile phones, smart watches).

The role of P.E. teachers is also critical. When developing and implementing the P.E. curriculum, the content needs to be focused in a pedagogical environment that goes beyond P.E. lessons and includes opportunities for everyday use. A special curriculum area related to this could be fitness education, which offers the opportunity to acquire knowledge and understanding of fitness and healthy lifestyle habits.^33^^,^^34^ It helps to develop the skills of short- and long-term planning and goal setting, measuring and self-tracking by building various fitness programmes, health-related exercises and sports activities. Unfortunately, this curriculum area is marginalized in many countries. In a recent study, Mercier *et al*.^35^ found that only 21% of high school P.E. teachers incorporated fitness concepts and knowledge during each class period.

A limitation of our work is that we did not gather data on the SES of respondents in the first data collection round. So, we cannot respond to such important questions whether there are inequalities regarding the level of activity during online P.E. classes. However, in the second round we observed that the lack of P.E. provision was more than two times higher among the highest socio-economic group compared to the lowest socio-economic group, but this difference disappeared after adjusting for the place of residence and the other variables.

### Panel: Recommendations for (re)building physical education

Based on our findings, as well as on the experience in our research group we recommend the following to realize all the potential benefits of P.E. in the post-pandemic era and to support recovery:

Policy level

- Teacher education programmes should continue to emphasize (i) the critical role of P.E. for students’ health and development, (ii) principles of inclusiveness, diversity and inclusivity and (iii) professional development for P.E. teachers should include how to deliver online P.E. lessons.
- Curriculum developers should revise the current content for P.E. and (i) adjust the content of P.E. to address the observed regression in neuromotor skills and physical literacy during COVID-19, and (ii) integrate more outdoor activities that take into consideration the cultural traditions of the country.
- Monitoring systems should be established to regularly assess the quality of P.E. lessons based on national standards.

Community level

- Stakeholders (particularly local sporting clubs) should align their efforts to ensure the necessary conditions, equipment and infrastructure to support quality P.E.

School level

- The allocated curriculum time for P.E. classes should not be replaced with other subjects and schools should aim for daily P.E. lessons.
- P.E. classes should provide at least 50% of class time in MVPA for students.
- P.E. should be delivered by qualified teachers.
- School principals should aim for a whole school approach as P.E. alone will not be enough to ensure children and youth meet the 60 mins of MVPA recommendation.
- Active school programmes should be tailored to the local context and implemented to support students in meeting the physical activity recommendation, and to reduce the time spent sedentary.

Classroom and teacher level

- Students should be active at a moderate- to vigorous-intensity for at least 50% of P.E. class time, and sedentary time should be reduced.
- High engagement of ‘all’ students should continue to be prioritized
- Teachers should encourage students to be active out of school.

Key points

- Physical education (P.E.) was far from optimal during distance learning compared with face-to-face classes.
- To support reversing the impact of the COVID-19 pandemic in children, current content of the P.E. curriculum has to be revised and number of P.E. lessons must be safeguarded or increased.
- Awareness of the critical role of P.E. for students’ health and development has to be strengthened along with the resources to support provision of quality P.E.
