# Correction to: Ringing the bell for quality P.E.: What are the realities of remote physical education?

**DOI:** 10.1093/eurpub/ckac190

**Published:** 2022-12-29

This is a correction to: Viktoria A Kovacs, Tamas Csanyi, Rok Blagus, Mirko Brandes, Gregor Starc, Paulo Rocha, Claude Scheuer, Anthony D Okely, Ringing the bell for quality P.E.: What are the realities of remote physical education?, *European Journal of Public Health*, Volume 32, Issue Supplement_1, September 2022, Pages i38–i43, https://doi.org/10.1093/eurpub/ckac082

In the originally published version of this manuscript, Viktoria Kovacs was identified as the corresponding author. The correct corresponding author is Tamas Csanyi (email: csanyi.tamas@tf.hu).

This error has been corrected online.

